# Identification of Salt Stress Responding Genes Using Transcriptome Analysis in Green Alga *Chlamydomonas reinhardtii*

**DOI:** 10.3390/ijms19113359

**Published:** 2018-10-26

**Authors:** Ning Wang, Zhixin Qian, Manwei Luo, Shoujin Fan, Xuejie Zhang, Luoyan Zhang

**Affiliations:** Key Lab of Plant Stress Research, College of Life Science, Shandong Normal University, No. 88 Wenhuadong Road, Jinan 250014, China; wangning_sdnu@163.com (N.W.); qianzhixin_sdnu@163.com (Z.Q.); luomanwei_sdnu@163.com (M.L.); fansj@sdnu.edu.cn (S.F.); zxjpublic@sohu.com (X.Z.)

**Keywords:** *Chlamydomonas reinhardtii*, salt stress, transcriptome analysis, impairment of photosynthesis, underpinnings of salt stress responses

## Abstract

Salinity is one of the most important abiotic stresses threatening plant growth and agricultural productivity worldwide. In green alga *Chlamydomonas reinhardtii*, physiological evidence indicates that saline stress increases intracellular peroxide levels and inhibits photosynthetic-electron flow. However, understanding the genetic underpinnings of salt-responding traits in plantae remains a daunting challenge. In this study, the transcriptome analysis of short-term acclimation to salt stress (200 mM NaCl for 24 h) was performed in *C. reinhardtii*. A total of 10,635 unigenes were identified as being differently expressed by RNA-seq, including 5920 up- and 4715 down-regulated unigenes. A series of molecular cues were screened for salt stress response, including maintaining the lipid homeostasis by regulating phosphatidic acid, acetate being used as an alternative source of energy for solving impairment of photosynthesis, and enhancement of glycolysis metabolism to decrease the carbohydrate accumulation in cells. Our results may help understand the molecular and genetic underpinnings of salt stress responses in green alga *C. reinhardtii*.

## 1. Introduction

Salinity is one of the most important abiotic stresses threatening agricultural productivity worldwide. Although plants have gradually evolved a series of adaptive molecular, physiology and biochemistry processes to respond to salinity stress, it could threaten 30% of cultivable soils by 2050 [1,2]. Understanding the molecular machineries of salt stress response in model plants of basal taxa, such as green algae, may contribute to finding the evolutionary cues of abiotic stress response in plants and developing salt-resistant crops with additional salt-responding traits [2,3,4,5,6,7,8,9].

Salt stress causes diverse impacts on plant growth by disturbing the osmotic/ionic balance and eliciting Na^+^ toxicity [9,10]. Under aquatic saline stress, a series of physical and biochemical processes are recruited by algae to respond to the damage caused by osmotic and ionic stresses, such as photosynthesis inhibition, macromolecular compound synthesis and homeostasis adjustment [6,10,11,12,13,14]. It has been reported that salt stress leads to decreased photosynthetic efficiency [15,16] which influences chlorophyll content in plant leaves [17,18]. In green algae, salt stress remarkably influences the structure and functions of the photosynthetic apparatus in *Scenedesmus obliquus* [19] and reduces the maximum quantum yield of photosystem II (PSII) in *Dunaliella salina* [20]. In alga *Botryococcus braunii*, metabolism of lutein was significantly enhanced under stress conditions [12].

*Chlamydomonas reinhardtii* is a free-living freshwater alga with unicellular vegetative cell. Previous studies exposed the *C. reinhardtii* strain 21 gr and CC-503 to salt stress and demonstrated the physiological and metabolic processes impacted by ionic toxicity and osmotic stress caused by salt damage [6,7,8,16,21]. Vega [22] demonstrated that 200 mM NaCl in the culture medium was highly toxic for *C. reinhardtii* productivity. The addition of NaCl immediately blocked the photosynthetic activity of the alga which partially recovered, after 1 h of treatment, remaining high during the following 24 h. However, after 24 h treatment with NaCl 200 mM, the intracellular catalase activity of the alga reached a 20-fold higher level than in the control cells. The physiological data indicate that saline stress induces in *C. reinhardtii* an increase of intracellular peroxide, which parallels a significant inhibition of the photosynthetic-electron flow. However, the related machineries of up-stream regulating and the triggering of appropriate cellular and physiological responses to cope with stress circumstances are still largely unknown.

Transcriptome sequencing is an effective strategy for detecting potential participants of stress response on a genome-wide scale. Hundreds of studies about salt stress responses in model plant *Arabidopsis thaliana* [23,24,25,26], crops *Oryza sativa* [23,27] and *Glycine max* [28], and in some halophytes (plants able to complete their life cycles under saline environments) have been widely conducted using sequencing technologies [7,29,30,31,32,33,34,35,36,37,38,39,40,41]. The integrations of genes’ spatio-temporal expression patterns and responding traits have helped to identify a large number of salt stress-related differentially expressed genes (DEGs) and mechanisms.

Keeping this in mind, the work presented here was carried out to explore the saline stress-responding mechanisms of *C. reinhardtii* by transcriptome sequencing of strains GY-D55 wild type. The aim of this study was to identify dys-regulated genes in *C. reinhardtii* cells under salt stress by RNA-seq, screen physiological and biochemical cues by gene ontology (GO) terms and MapMan functional enrichment analyses, and investigate the physiological adaptions and cellular regulatory networks for salt stress responding.

## 2. Results

### 2.1. Transcriptome Profiling of C. reinhardtii 

After sequencing with the Illumina HiSeq X platform, a total of 56,438,218, 72,853,712, 47,551,786, 56,962,722, 52,926,804 and 55,998,748 high-quality pair-end reads were obtained from three control and three salt stress treated samples of *C. reinhardtii* (Table 1), respectively. *De novo* transcriptome assembly generated 91,242 unigenes, with an average length of 2691 nt and N50 of 4554. On average, 90.66% of the reads from six samples were mapped to the reference genome (Table 1). The assembled transcriptome information of *C. reinhardtii* is shown in Supplementary Figure S1.

### 2.2. Functional Annotations of Unigenes

**Figure 1:**
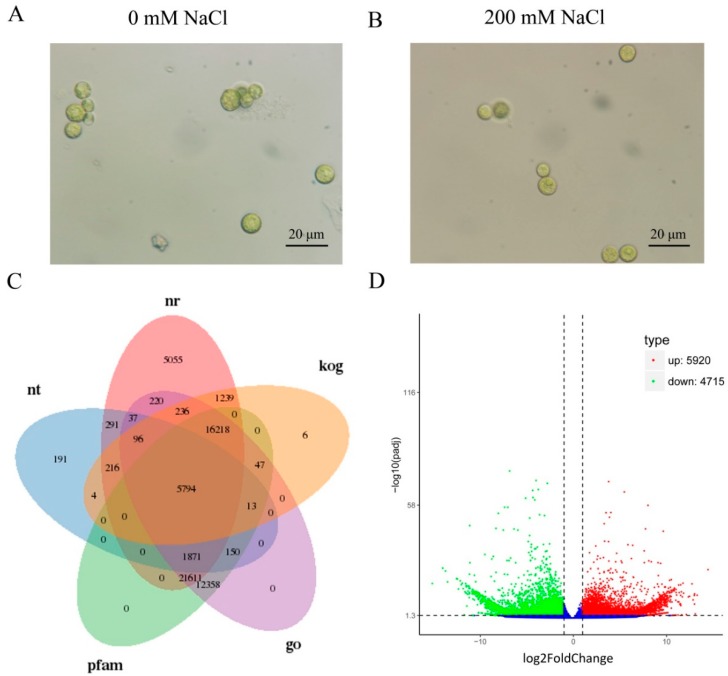
(**A**) The morphology of *C. reinhardtii* cells without addition of NaCl. (**B**) The morphology of *C. reinhardtii* cells under 200 mM NaCl treatment. (**C**) Venn diagram of functional annotations of unigenes in nt (NCBI non-redundant protein sequences), nr (NCBI non-redundant protein sequences), kog (Clusters of Orthologous Groups of proteins), go (Gene Ontology) and pfam (Protein family) databases. (**D**) Expression patterns of differentially expressed genes (DEGs) identified between 200 mM NaCl treated and control. S_200 indicated cells under 200 mM NaCl stressed condition for 24 h; C_0 indicated cells cultured under control condition. Red and green dots represent DEGs, blue dots indicate genes that were not differentially expressed. In total, 10,635 unigenes were identified as DEGs (padj < 0.05) between S_200 and C_0, including 5920 upregulated genes and 4715 downregulated genes.

Similarity searches were performed to annotate unigenes against different databases using BLASTX. For *C. reinhardtii*, 65,679 (71.98%) unigenes were annotated in at least one database (Figure 1C and Supplementary Figure S1). A total of 52,884 (57.96%) and 58,062 (63.63%) unigenes showed similarity to sequences in NR and PFAM databases with an E-value threshold of 1 × 10^−5^. About 58,651 (64.28%) unigenes were annotated in the GO database by Blast2GO v2.5 with an E-value cutoff of 1 × 10^−6^ (Figure 1C and Supplementary Figure S1). Unigenes of the *C. reinhardtii* were assigned to *C. reinhardtii* and *A. thaliana* gene IDs for GO annotation mapping and TFs/PKs perdition. By sequence alignment, a total of 48,158 unigenes were aligned to *C. reinhardtii* PLAZA genome genes. A total of 54,509 unigenes were assigned to TAIR10 locus IDs by BLASTP with an E-value cutoff of 1 × 10^−5^ and classified into GO categories for GO analysis (Supplementary Table S1).

### 2.3. Differently Expressed Genes (DEGs) Calculation 

To evaluate the relative level of gene expression in *C. reinhardtii* under control or salt stress treatment, the FPKM values were calculated based on the uniquely mapped reads. The FPKM distributions of unigenes in six samples are shown in Supplementary Figure S2. The FPKM value for genes detected in six samples ranged from 0 to 40,486.05, with mean value of 7.08. By comparative analysis, a part of the genes was observed to be differently expressed in 200 Mm NaCl treated samples: 5920 unigenes were calculated as up-regulated in salt treated samples and 4715 filtered as down-regulated genes with the cutoff of padj < 0.05 and |log2(foldchange)| > 1 (Supplementary Table S2).

The most significantly dysregulated 30 genes are recorded in Table 2. The most significantly upregulated unigenes included RNA recognition motif containing gene Cluster-2749.47186 (log2FoldChange [L_2_fc] = 3.894), “transcription, DNA-templated” participating gene Cluster-2749.64181 (L_2_fc = 5.573) and “potassium ion transport” gene Cluster-2749.61362 (L_2_fc = 8.112) (Table 2 and Supplementary Table S2). Downregulated unigenes, included “chlorophyll metabolic process” related gene Cluster-2749.44503 (L_2_fc = −8.623) with the lowest *p*-value, “proteolysis” related gene Cluster-2749.61923 (L_2_fc = −6.748) and “regulation of transcription, DNA-templated” participating gene Cluster-2749.45379 (L_2_fc = −3.663).

### 2.4. GO Enrichment of DEGs

For uncovering the differences of molecular mechanisms of *C. reinhardtii* under salt stress, the DEGs were then characterized with GO databases. A total of 353 biological processes (BP) terms were enriched by the 5920 up-regulated unigenes, like “oxidation-reduction process” (GO:0055114), “response to cadmium ion” (GO:0046686) and “response to salt stress” (GO:0009651) (Table 3; Supplementary Table S3). The 4715 down-regulated genes were calculated enriched in 313 BP terms, as “photosynthesis, light harvesting in photosystem I” (GO:0009768), “chlorophyll biosynthetic process” (GO:0015995) and “isoleucine biosynthetic process” (GO:0009097) (Table 3; Supplementary Table S3).

### 2.5. MapMan Enrichment of DEGs

A more specific comparison of metabolic and regulatory pathways was conducted using MapMan. A total of 5920 up- and 4715 down-regulated genes were assigned to 1334 and 1050 homologs in *Arabidopsis thaliana*, respectively. Consequently, these uniquely expressed genes were mapped to 797 pathways by MapMan, of which, 22 pathways were filtered enriched by the dysregulated genes with the cutoff *p*-value < 0.05 (Figure 3A; Supplementary Table S4). The expression of genes implicated in “TCA/org. transformation.TCA”, “Tetrapyrrole synthesis”, “Starch” and “Sucrose” were over-expressed in *C. reinhardtii*, while those genes involved in “PS.lightreaction”, “PS.lightreaction.photosystem I” and “PS.lightreaction.photosystem I.LHC-I” were down-regulated in *C. reinhardtii* during salt stress responding (Figure 3A).

### 2.6. KEGG Enrichment of DEGs

**Figure 2:**
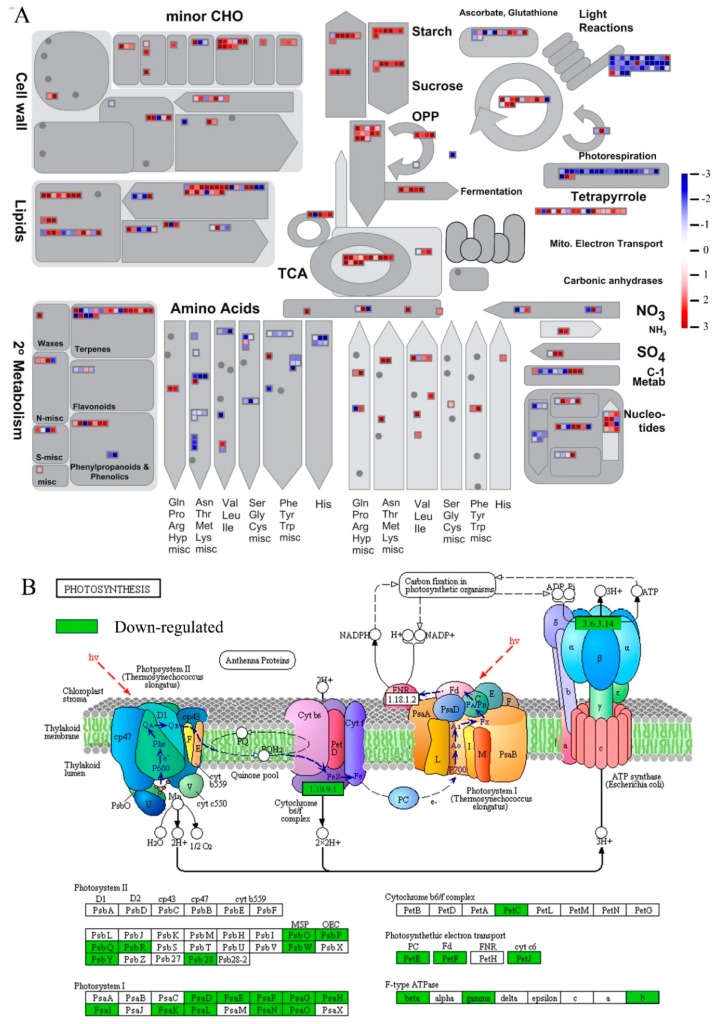
(**A**) Global view of differently expressed genes (DEGs) involved in diverse metabolic pathways. DEGs genes were selected for the metabolic pathways analysis using the MapMan software (3.5.1 R2). The colored boxes indicate the Log_2_ of expression ratio of DEGs genes. The dys-regulated unigenes were assigned to 1334 and 1050 homologs in Arabidopsis, respectively. These genes were mapped to 797 pathways by MapMan, of which, 22 pathways were filtered enriched by the dys-regulated genes with the cutoff *p*-value < 0.05. (**B**) The KEGG pathways (ko00195) “photosynthesis” mapped with 44 down-regulated unigenes. The down-regulated genes are marked by a green frame. The black solid line with a black arrow means molecular interaction or relation; the black dash line with a black arrow means indirect link or unknown reaction; the red dash line with a red arrow stands for the light quanta.

To gain a deeper insight into the regulation of photosynthesis underlying salt stress response, down-regulated unigenes involved in “photosynthesis” KEGG pathways (ko00195) were mapped and shown in Figure 2B. Orthologs of 44 genes annotated in this pathway were filtered as down-regulated in the NaCl treated samples in the green alga, such as, photosystem II oxygen-evolving enhancer protein PSBO Cluster-2749.35825 (L_2_fc = −2.5458) and Cluster-2749.43661 (L_2_fc = −2.1558), cytochrome b6-f complex iron-sulfur subunit PETC, Cluster-2749.42943 (L_2_fc = −2.7088), and F-type H+-transporting ATPase subunit ATPF0A, Cluster-2966.0 (L_2_fc = −3.1245) (Figure 2B; Supplementary Table S2).

### 2.7. The Differentially Expressed TFs and PKs

Among the expressed unigenes, 2050 and 1624 sequences were assigned to 45 TF families and 78 PK families, respectively (Supplementary Table S5). Of the TF families, MYB family had the largest number of upregulated genes (16 unigenes), including MYB109 ortholog unigenes Cluster-2749.35807 (L_2_fc = 2.5798) and Cluster-2749.70085 (L_2_fc = 1.3722). In contrast, SET family had the largest number of downregulated genes (16 unigenes). Of the PKs families, TKL-Cr-3 family was uncovered to contain the largest number of upregulated genes. By comparison, CAMK_CDPK and Group-Cr-2 family contained the largest number of downregulated unigenes (Supplementary Table S5).

### 2.8. Real-Time Quantitative PCR Validation

**Figure 3:**
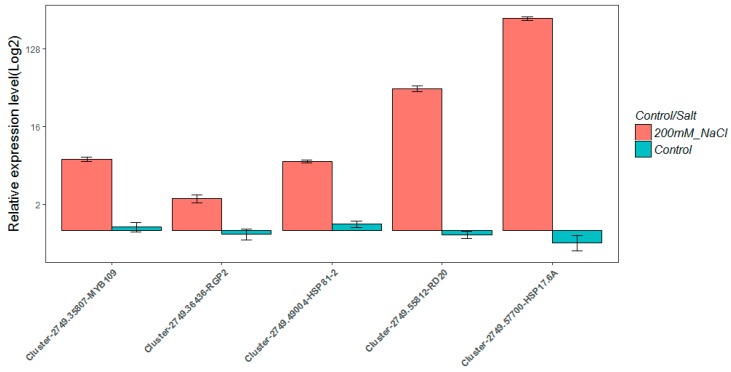
Real-time PCR verification of five up-regulated genes in *C. reinhardtii*. The red bars represent the qPCR results of samples under salt stress condition, while the corresponding blue bars represent the results of control samples. The individual black bars, representing the qPCR data, are the means ± SD of nine measurements (three technical replicates each for three biological samples).

To verify the RNA-seq results, an alternative strategy was selected for the upregulated unigenes. In total, five over-expressed unigenes were randomly selected for validation by qRT-PCR using the same RNA samples that were used for RNA-seq. Primers were designed to span exon-exon junctions (see Supplementary Table S6 and Figure S3). In most cases, the gene expression trends were similar between these two methods; the result is shown in Figure 3. The ortholog of cytosolic small heat shock protein encoding genes HSP17.6A, Cluster-2749.57700, which was detected by RNA-Seq as up-regulating genes in the salt treated samples (L_2_fc = 9.76), was also detected to be significantly over-expressed by qRT-PCR method (Figure 3).

## 3. Discussion

Salinity is one of the major environmental factors threatening crop productivity and plant growth worldwide [2,9,42]. Due to the complexity of abiotic stress-responding processes, although several hundreds of salt-responding genes have been reported in plants, understanding the genetic underpinnings of salt-responding traits in plantae remains a daunting challenge. The model alga *C. reinhardtii*, which contains one large cup-shaped chloroplast, has the ability to adapt rapidly to changing environmental conditions, such as high salinity, via the generation of novel traits [8,14,43,44]. Given previous results from analysis of salt stress in *C. reinhardtii* and other plants, we analyzed the Illumina RNA-seq data from this alga grown in BG-11 medium with the addition of 200 mM NaCl and analyzed in triplicate after 24 h of incubation [16,22]. In this study, a total of 5920 and 4715 unigenes were identified as up- and down-regulated genes in *C. reinhardtii* under salt stress by RNA-seq. Our study found some molecular cues for reducing the negative effects due to ionic/osmotic toxicity and photosynthesis impairment under saline conditions in *C. reinhardtii*.

Previous studies discovered that the cell density of *C. reinhardtii* cells obviously reduced when stressed by NaCl [8,14,16,21,22]. Neelam et al. demonstrated that at the morphological level, 150 or 200 mM NaCl salt stress led to palmelloid morphology, flagellar resorption, reduction in cell size, and slower growth rate in *C. reinhardtii* [21]. It should be noted that dead and dying cells have dys-regulated mRNA and contribute to transcript levels under saline stress. In our study, programmed cell death (PCD) in the *C. reinhardtii* cell was found with PCD-regulating proteins being significantly up-regulated, e.g., condensin complex subunit (Cluster-2749.11751: L_2_fc = 9.368; Cluster-2749.12889: L_2_fc = 7.766), sucrose-phosphatase 1 (Cluster-2749.35394: L_2_fc = 4.304) and stress tolerance related fibrillin family member (Cluster-2749.70284: L_2_fc = 1.920).

Saline stress leads to the overproduction of reactive oxygen species (ROS) in plants which are highly reactive and toxic and cause damage to lipids, carbohydrates, proteins and DNA which ultimately results in oxidative stress [8,9,14,45]. The accumulation of ROS also influences the expression of a number of genes and therefore controls many processes, such as growth, cell cycle, PCD, secondary stress responses and systemic signaling [8,9,14,45]. The excess Na^+^ and oxidative stress in the intracellular or extracellular environment activates the acytoplasmic Ca^2+^ signal pathway for regulating an osmotic adjustment or homeostasis regulating of salt stress responses [24,29,39,46,47,48,49,50,51]. In our study, calcium-related pathway in the *C. reinhardtii* cell was found with several calcium ion binding proteins being significantly upregulated, e.g., peroxygenase 3 (Cluster-2749.55812: L_2_fc = 10.431; Cluster-2749.59997: L_2_fc = 7.680) and calreticulin (Cluster-2749.35394: L_2_fc = 3.082).

The short-term (within 48 h) acclimation to salt stress in *C. reinhardtii* involves activation of phospholipid signaling, leading to the accumulation of phosphatidic acid (PA), which is a lipid second messenger in plant and animal systems [52,53,54]. In the case of *C. reinhardtii*, incubation in 150 mM NaCl leads to a three- to four-fold rise of PA levels within minutes [52,55]. Lysophosphatidic acid (LPA) has also been shown to accumulate in this alga under salt stress, with the dose-dependent response reaching a maximum at 300 mM NaCl [55,56]. In this study, soluble lysophosphatidic acid acyltransferase (Cluster-2749.8269: L_2_fc = 9.126; Cluster-2749.9895: L_2_fc = 8.720) was found to be significantly up-regulated in salt stress treated samples, which indicated the potential role of this gene in maintaining the lipid homeostasis by regulating PA under saline stress [55]. Further, analysis of glycerophospholipid metabolism pathways showed that the alga cells had significant up-regulation of FAD (flavin adenine dinucleotide)-dependent oxidoreductase family protein (Cluster-2749.52046; L_2_fc = 2.695) that involves storing lipid catabolism and glycerol assimilation, and in glycerol-3-phosphate shuttle, which transports reduced power from cytosol to mitochondrion [8]. This suggests that the intracellular glycerol pool in *C. reinhardtii* cells likely increased as a response to salt stress, similar to what has been shown for the green alga *Dunaliella tertiolecta* [57,58].

Requirement of energy to maintain ion homeostasis is the major metabolic impact of salt stress. The reduction of oxidative stress and osmotic stress, and the up-regulation of heatshock proteins were speculated to aid protein renaturation and recover homeostasis [59,60,61]. In this study, the stress response is apparent in the *C. reinhardtii* cells with significant up-regulation of genes involved in oxidative/osmotic stress reduction process including glyceraldehyde-3-phosphate dehydrogenase C subunit 1 (Cluster-2749.27769: L_2_fc = 1.930) and fumarase 1 (Cluster-2749.35832: L_2_fc = 8.306). In bacterium *Escherichia coli*, trehalose is synthesized as a compatible solute and enables cells to exclude toxic cations and to acclimate to high concentrations of salt in the growth medium [62]. For maize, trehalose has helped to reduce the negative effects of saline stress as an osmoprotectant [63]. In our study, enzymes involved in trehalose synthesis significantly up-regulated, e.g., trehalose-6-phosphatase synthase S8 (Cluster-2749.17684: L_2_fc = 6.453) and trehalose-6-phosphate synthase (Cluster-2749.61951: L_2_fc = 1.123). These results indicated the potential underpinnings for these to maintain homeostasis in *C. reinhardtii* under saline conditions.

In plants, saline stress generally causes ion injury and osmotic stress, which interferes with numerous biochemical and physiological processes, including energy metabolism pathways such as photosynthesis [26,36,64,65] and photorespiration [8]. Previous pigment analyses have demonstrated that photosystem I-light harvesting complexes (LHCs) are damaged by ROS at high salt conditions, and PSII proteins involved in oxygen evolution are impaired [21,45]. In our study, impairment of photosynthesis in the *C. reinhardtii* cell population was found, with several photosystem I-light harvesting complex (LHC) proteins being significantly down-regulated (Figure 2B), e.g., photosystem I light harvesting complex gene LHCA2 (Cluster-2749.32743: L_2_fc = −4.74; Cluster-2749.52511: L_2_fc = −3.28), LHCA3 (Cluster-2749.43129: L_2_fc = −6.583) and LHCA5 (Cluster-2749.40312: L2fc = −11.375; Cluster-2749.34085: L_2_fc = −6.553). Further, we found most of the chloroplast encoded transcripts (e.g., PsaA, B, C, J, M) in photosystem I (PSI) were relatively unchanged in level while the nuclear genes (e.g., PsaD, E, G, F, H) down-regulated under saline conditions (Figure 2B). Existing studies have demonstrated the usage of acetate in the medium as alternative source of energy to compensate for the lowered efficiency in photosynthesis [66]. Consistent with this view, we found that acetyl-CoA synthetase (Cluster-2749.60516: L_2_fc = 5.144; Cluster-2749.25511: L_2_fc = 2.495), which combines acetate and CoA to form acetyl-CoA, was significantly up-regulated in the alga cells under saline conditions. In this study, a significant down-regulation was found in a key enzyme of the glyoxylate cycle—isocitrate lyase (ICL, [Cluster-2749.51492; L_2_fc = −3.119]) [8,45,66,67]—which catalyzes the cleavage of isocitrate to succinate and glyoxylate. Together with malate synthase, ICL bypasses the two decarboxylation steps of the tricarboxylic acid cycle (TCA cycle) [8]. The spatio-temporal expression patterns of genes suggest that in alga cells acetyl-CoA is introduced into energy generation pathways for salt stress responses.

Glycolysis is considered to play an important role in plant development and adaptation to multiple abiotic stresses, such as cold, salt, and drought. It is the key respiratory pathway for generating ATP and carbohydrates metabolites [50,68,69,70,71,72,73]. In our work, salt stress significantly increased the expression of genes participating in the metabolism of main carbohydrates, such as starch, sucrose, soluble sugar and glucose (Figure 2A). For example, 31 genes of “glycolytic process” (GO:0006096) over-expressed during salt stress responding, including plastidic pyruvate kinase PKP-ALPHA (Cluster-2749.14688: L_2_fc = 8.53) and PKP-BETA1 (Cluster-2749.26182: L_2_fc = 3.68). This is consistent with Zhong et al. [68], who reported salt stress significantly increased the main carbohydrate contents of cucumber leaves [53]. Carbohydrates are involved not only in osmotic adjustment, but also can be used as protective agents for homeostasis regulating during salt stress tolerance [24,30,39,48,69,70,74,75,76,77]. Given that salt injury caused the destruction of photosynthesis, which might inhibit transport of carbohydrate and accumulate excess starch or sucrose, we speculate *C. reinhardtii* enhanced glycolysis metabolism to decrease carbohydrate accumulation in cells, which would promote the respiratory metabolism and mitochondrial electron transport, thus reducing the effects of ionic toxicity and osmotic stress caused by salt damage.

## 4. Materials and Methods

### 4.1. Chlamydomonas Material Preparation, Salt Stress Treatment and RNA Extraction

The *C. reinhardtii* strain GY-D55 wild type from LeadingTec (Shanghai, China) were grown in 150 mL of BG11 media, and placed on a shaking table with 120 rpm and maintained at light (16 h)/dark (8 h) at 23 °C, with an illumination of 100 μmol m^−2^·s^−1^. The density of cell cultures was determined by using the blood cell counting plate, with each value being the means of 6 repeats. Under this condition, *C. reinhardtii* cells were grown in BG11 for 14 d.

The methods published by Zhao [16] and Vega [22] were referenced for NaCl treatment in this study. A total of 50 mL medium with 800 mM NaCl was added to the 150 mL culture medium on a shaking table for finishing 200 mM NaCl treatment, the added NaCl was rapidly diluted, and then the pH value was adjusted to 7.0. A parallel set of cells that were unexposed to NaCl stress conditions and cultured in medium served as the experimental control. A total of 50 mL medium without NaCl was added into the control group. Each treatment had 3 repeats. For 24 h, 200 mM NaCl treatment significantly affected the cellular physiology of the alga, such as its photosynthetic and intracellular catalase activity; in this study, the culture time for *C. reinhardtii* under salt stress was 24 h.

After 24 h, 100 mL cell culture medium was extracted from the NaCl treated and control culture bottles, respectively. The collected cells were centrifuged at 3000× *g* for 5 min, and the collected cells were resuspended in 25 mL RNAlater (Ambion, Shanghai, China) solution for RNA extraction. The cells of each repeat were mixed and total RNAs were extracted separately using the TRIzol Reagent (Invitrogen, Carlsbad, CA, USA) following the manufacturer’s procedures. RNA quality was assessed using the RNA Nano 6000 Assay Kit of the Agilent Bioanalyzer 2100 system (Agilent Technologies, Santa Clara, CA, USA) and the NanoDrop 2000 spectrophotometer (Thermo Scientific, Wilmington, NC, USA).

### 4.2. Illumina Library Construction and Sequencing

A total amount of 1.5 μg RNA per sample was used as input material for the RNA sample preparations. Sequencing libraries were generated using the NEBNext^®^ Ultra^TM^ RNA Library Prep Kit for Illumina^®^ (NEB, San Diego, CA, USA) by following manufacturer’s procedures, and index codes were added to attribute sequences to each sample. Briefly, mRNA was purified from total RNA using poly-T oligo-attached magnetic beads. The random hexamer primer and M-MuLV Reverse Transcriptase (RNase H^−^) were used to synthesize the first strand cDNA and the DNA Polymerase I and RNase H were used for second strand cDNA synthesis. Fragments of 150~200 bp cDNA were purified with the AMPure XP system (Beckman Coulter, Beverly, MA, USA). Then, 3 μL USER Enzyme (NEB, USA) was used with size-selected, adaptor-ligated cDNA at 37 °C for 15 min followed by 5 min at 95 °C. Then, PCR was performed with Phusion High-Fidelity DNA polymerase, Universal PCR primers and Index (X) Primer. Ten cycles were used for PCR enrichment. Finally, PCR products were purified (AMPure XP system) and library quality was assessed on the Agilent Bioanalyzer 2100 system. The clustering of the index-coded samples was performed on a cBot Cluster Generation System using TruSeq PE Cluster Kit v3-cBot-HS (Illumia, San Diego, CA, USA) according to the manufacturer’s instructions. After cluster generation, the library preparations were sequenced on an Illumina HiSeq X platform (Illumina, San Diego, CA, USA), according to the manufacturer’s procedures. All genetic data have been submitted to the NCBI Sequence Read Archive (SRA) database (https://www.ncbi.nlm.nih.gov/sra), SRA accession: PRJNA490089.

### 4.3. De Novo Transcriptome Assembling and Unigene Annotation

RNA sequencing and de novo transcriptome assembling were conducted to create reference sequence libraries for *C. reinhardtii*. The RNA sample of each repeat was sequenced separately. cDNA library construction and Illumina pair-end 150 pb sequencing (PE150) were performed at Novogene Co., Ltd. (Shanghai, China), according to instructions provided by Illumina Inc. Reads containing adapter, ploy-N and low-quality reads were removed from raw data for obtaining clean reads. The filtered high-quality reads were used for transcriptome assembling by the Trinity software with default parameters [78]. Clean datasets of 6 samples were pooled for de novo assembling and comprehensive sequence library construction. The Basic Local Alignment Search Tool (BLAST) searches of de novo assembled sequences against public databases (NR, NT, Swiss-Prot, Pfam, KOG/COG, Swiss-Prot, KEGG Ortholog database and Gene Ontology) with an E-value threshold of 10^−10^ were used for unigenes’ annotation.

### 4.4. Calculation and Comparison of Unigene Expression 

The independent transcripts libraries of 3 repeats under NaCl treatment conditions and 3 under control conditions were generated for *C. reinhardtii* by a PE150 sequencing analysis. The clean reads were aligned to the de novo assembled transcriptome and estimated by the RSEM [79] method. Gene expression levels were calculated by the fragment per kilobase of exon model per million mapped reads (FPKM) method. DESeq2 [80] was used to compare the expression levels between NaCl treated and control samples with an cutoff of adjusted *p*-value (padj) < 0.05 and |log2(foldchange)| > 1.

### 4.5. Gene Ontology (GO), Transcription Factors (TFs) and Protein Kinases (PKs) Prediction

The unigenes were transferred to the *C. reinhardtii* and *A. thaliana* gene IDs by using sequence similarity searching analysis against the genome of *C. reinhardtii* (ftp://ftp.psb.ugent.be/pub/plaza/plaza_public_dicots_04/Fasta/cds.all_transcripts.cre.fasta.gz) and *A. thaliana* (ftp://ftp.psb.ugent.be/pub/plaza/plaza_public_dicots_04/Fasta/cds.all_transcripts.ath.fasta.gz) with an E-value cutoff of 10^−5^. The classifications of TFs and PKs of *C. reinhardtii* were downloaded from the iTAK database (http://bioinfo.bti.cornell.edu/cgi-bin/itak/index.cgi) [81]. The GO functional annotations file of *A. thaliana* was downloaded from Gene Ontology database (submitted 5 June 2018, http://geneontology.org/gene-associations/gene_association.tair.gz). The TFs and PKs of *C. reinhardtii* genes were transferred to their hit unigenes and the GO functional annotations of *A. thaliana* genes were assigned to their ortholog unigenes in *C. reinhardtii*.

### 4.6. GO, KEGG and MapMan Annotation and Enrichment

The GO enrichment analysis for DEGs of *C. reinhardtii* was performed by the topGO package of R. KEGG [82] is a database resource for understanding high-level functions and utilities of the biological system, such as the cell, the organism and the ecosystem, from molecular-level information, especially large-scale molecular datasets generated by genome sequencing and other high-throughput experimental technologies (http://www.genome.jp/kegg/). We used KOBAS [83] software to test the statistical enrichment of differential expression genes in KEGG pathways. MapMan (version 3.5.1 R2) [84] was also used to annotate the DEGs onto metabolic pathways. The DEGs of *C. reinhardtii* unigene IDs were transferred to the Arabidopsis Information Resource (TAIR) locus IDs during the MapMan analysis.

### 4.7. Real-Time Quantitative PCR (qRT-PCR) Verification

Real-time quantitative PCR (qRT-PCR) was performed to verify the expression patterns revealed by the RNA-seq analysis. The purified RNA of samples under salt stress and control conditions were treated with DNaseI and converted to cDNA using the PrimeScript RT Reagent Kit with gDNA Eraser (Takara, Dalian, China) according to the manufacturer’s procedures. Five up-regulated unigenes in *C. reinhardtii* were selected for the qRT–PCR assay, including Cluster-2749.49004 (ortholog of HSP81-2), Cluster-2749.57700 (ortholog of HSP17.6A), Cluster-2749.55812 (ortholog of RD20), Cluster-2749.36436 (ortholog of RGP), and Cluster-2749.35807 (ortholog of MYB109). Gene-specific qRT–PCR primers (18–20 bp) (Table S6) were designed using Premier 5.0 software. qPCR was performed using SYBR Green qPCR Master Mix (DBI, Ludwigshafen, Germany) in ABI7500 Real-Time PCR System (ABI, Waltham, MA, USA). Three replicates were performed, and the amplicons were used for melting curve analysis to evaluate the amplification specificity. Relative gene expression was quantified using the 2^−(ΔΔ*C*t)^ method [85]. Ortholog of the *A. thaliana* housekeeping GTP binding Elongation factor Tu family member AT5G60390 in *C. reinhardtii* (Cluster-2749.43263) was used to normalize the amount of template cDNA added in each reaction.

## 5. Conclusions

We performed a transcriptome analysis of short-term acclimation to salt stress (200 mM NaCl for 24 h) in *C. reinhardtii*. In total, 10,635 unigenes were identified as differentially expressed in *C. reinhardtii* under salt stress by RNA-seq, including 5920 that were up- and 4715 that were down-regulated. A series of molecular cues were screened by GO terms, MapMan and KEGG functional enrichment analyses, which were identified as potential mechanisms for salt stress responses. These mainly include maintaining the lipid homeostasis by regulating phosphatidic acid, acetate being used as an alternative source of energy for solving impairment of photosynthesis and enhancement of glycolysis metabolism to decrease the carbohydrate accumulation in cells. Our results may help understand the molecular and genetic underpinnings of salt responding traits in green alga *C. reinhardtii*.

## Figures and Tables

**Table 1:** Summary of mapping transcriptome reads to reference sequence.

Sample Name	Sample Description	Total Reads	Total Mapped	Ratio of Mapped Reads
C_0_1	Control replication 1	56,438,218	51,454,456	91.17%
C_0_2	Control replication 2	72,853,712	66,008,290	90.60%
C_0_3	Control replication 3	47,551,786	43,268,544	90.99%
S_200_1	Salt stress replication 1	56,962,722	51,633,614	90.64%
S_200_2	Salt stress replication 2	52,926,804	47,815,814	90.34%
S_200_3	Salt stress replication 3	55,998,748	50,507,824	90.19%

**Table 2:** Top30 dysregulated genes in *C. reinhardtii* under 200 mM NaCl treated and control conditions.

Gene_ID	L_2_fc	*p*val	BP Description
Up-regulated			
Cluster-2749.47186	3.894	3.77 × 10^−75^	
Cluster-2749.64181	5.573	1.55 × 10^−69^	transcription, DNA-templated
Cluster-2749.61362	8.112	1.95 × 10^−62^	potassium ion transport
Cluster-2749.33332	4.129	1.19 × 10^−58^	signal transduction
Cluster-2749.48242	3.610	1.64 × 10^−58^	
Cluster-2749.21356	3.975	4.34 × 10^−56^	
Cluster-2749.37168	3.413	1.00 × 10^−52^	
Cluster-2749.23874	7.849	5.01 × 10^−50^	lipid metabolic process
Cluster-2749.57700	9.756	9.76 × 10^−49^	iron-sulfur cluster assembly
Cluster-2749.59287	3.459	1.42 × 10^−43^	cell adhesion
Cluster-2749.53252	3.877	2.36 × 10^−43^	pathogenesis
Cluster-2749.49912	5.957	1.07 × 10^−41^	lipoprotein metabolic process
Cluster-2749.84953	6.468	2.29 × 10^−41^	
Cluster-2749.82821	2.504	5.20 × 10^−41^	regulation of protein kinase activity
Cluster-2749.3203	7.706	1.83 × 10^−38^	
Down-regulated			
Cluster-2749.44503	−8.623	4.01 × 10^−178^	chlorophyll metabolic process
Cluster-2749.61923	−6.748	6.07 × 10^−81^	proteolysis
Cluster-2749.38883	−3.906	7.54 × 10^−76^	
Cluster-2749.44595	−2.699	3.50 × 10^−74^	metabolic process
Cluster-2749.45379	−3.663	6.53 × 10^−71^	regulation of transcription, DNA-templated
Cluster-2749.49076	−4.268	2.29 × 10^−70^	chlorophyll biosynthetic process
Cluster-2749.44117	−4.239	1.30 × 10^−66^	oxidation-reduction process
Cluster-2749.42573	−5.023	3.04 × 10^−66^	protein glycosylation
Cluster-2749.32226	−4.043	2.67 × 10^−65^	proteolysis
Cluster-2749.45636	−7.283	1.98 × 10^−61^	
Cluster-2749.44732	−6.934	2.08 × 10^−61^	
Cluster-2749.49721	−7.951	3.18 × 10^−58^	
Cluster-2749.65261	−3.524	1.91 × 10^−57^	
Cluster-2749.36258	−2.996	5.32 × 10^−57^	
Cluster-2749.43872	−4.589	1.10 × 10^−55^	cell adhesion

Note: Top30 dysregulated genes with the lowest *p*-value (*p*val) are represented; L_2_fc indicates the log2FoldChange of genes differently expressed in 200 mM NaCl treated samples and control samples; BP Description means descriptions of genes’ potential participating biological process predicted by sequence similarity search.

**Table 3:** Top30 biological processes enriched by the up- and down-regulated genes.

GO ID	GO Term	Annotated Gene Number	Enriched Gene Number	*p*-Value
Up-Regulated				
GO:0008150	biological process	33682	2820	1.00 × 10^−30^
GO:0055114	oxidation-reduction process	3653	385	2.90 × 10^−27^
GO:0046686	response to cadmium ion	1317	159	3.40 × 10^−18^
GO:0042542	response to hydrogen peroxide	189	41	1.10 × 10^−15^
GO:0009408	response to heat	717	122	1.40 × 10^−15^
GO:0051259	protein oligomerization	109	25	7.50 × 10^−12^
GO:0010090	trichome morphogenesis	131	26	4.60 × 10^−10^
GO:0009414	response to water deprivation	668	79	6.70 × 10^−10^
GO:0009651	response to salt stress	1488	143	3.90 × 10^−09^
GO:0043335	protein unfolding	39	14	1.80 × 10^−08^
GO:0016036	cellular response to phosphate starvation	262	40	2.40 × 10^−08^
GO:0010030	positive regulation of seed germination	85	20	6.50 × 10^−08^
GO:0030866	cortical actin cytoskeleton organization	31	12	7.20 × 10^−08^
GO:0016477	cell migration	31	12	7.20 × 10^−08^
GO:0045010	actin nucleation	31	12	7.20 × 10^−08^
Down-Regulated				
GO:0008150	biological process	33682	2018	1.00 × 10^−30^
GO:0009768	photosynthesis, light harvesting in photosystem I	87	46	1.00 × 10^−30^
GO:0009645	response to low light intensity stimulus	72	37	1.00 × 10^−30^
GO:0015995	chlorophyll biosynthetic process	242	54	4.40 × 10^−29^
GO:0009644	response to high light intensity	393	71	6.70 × 10^−22^
GO:0006412	translation	1779	179	3.80 × 10^−16^
GO:0009409	response to cold	978	103	5.30 × 10^−16^
GO:0009269	response to desiccation	41	18	1.00 × 10^−14^
GO:0009769	photosynthesis, light harvesting in photosystem II	36	17	1.30 × 10^−14^
GO:0010218	response to far red light	101	25	8.70 × 10^−14^
GO:0006364	rRNA processing	742	89	2.10 × 10^−12^
GO:0010114	response to red light	159	28	5.90 × 10^−11^
GO:0015979	photosynthesis	853	137	2.40 × 10^−10^
GO:0009097	isoleucine biosynthetic process	53	16	2.60 × 10^−10^
GO:0009099	valine biosynthetic process	43	14	1.10 × 10^−09^

## Supplementary Materials

The following are available online at http://www.mdpi.com/1422-0067/19/11/3359/s1, Figure S1: The assembled transcriptome information of *C. reinhardtii*, Figure S2: The FPKM density distribution of *C. reinhardtii*, Table S1: Unigenes of *C. reinhardtii* annotated in *A. thaliana* genome by BLASTX analysis, Table S2: Information of the 5920 up- and 4715 down-regulated unigenes in *C. reinhardtii*, Table S3: The Gene Ontology (GO) enrichment results of the dys-regulated genes in *C. reinhardtii*, Table S4: The MapMan pathways enrichment results of the dys-regulated genes in *C. reinhardtii*, Table S5: Information of the differently expressed transcription factors (TFs) and protein kinases (PKs), Table S6: Information of the qRT–PCR primers.

## Abbreviations

DEGs	differentially expressed genes
TFs	transcriptional factors
PKs	protein kinases
GO	gene ontology
FPKM	fragment per kilobase of exon model per million mapped reads
qRT-PCR	real-time quantitative PCR
BP	biological processes
PSI	photosystem I
PSII	photosystem II
PCD	programmed cell death
ROS	reactive oxygen species
PA	phosphatidic acid
