# Supplementary figures and images for: Identification of Salt Stress Responding Genes Using Transcriptome Analysis in Green Alga Chlamydomonas reinhardtii
Source: Int J Mol Sci. 2018 Oct 26;19(11):3359. doi: 10.3390/ijms19113359 (PMC6274750; doi:10.3390/ijms19113359)

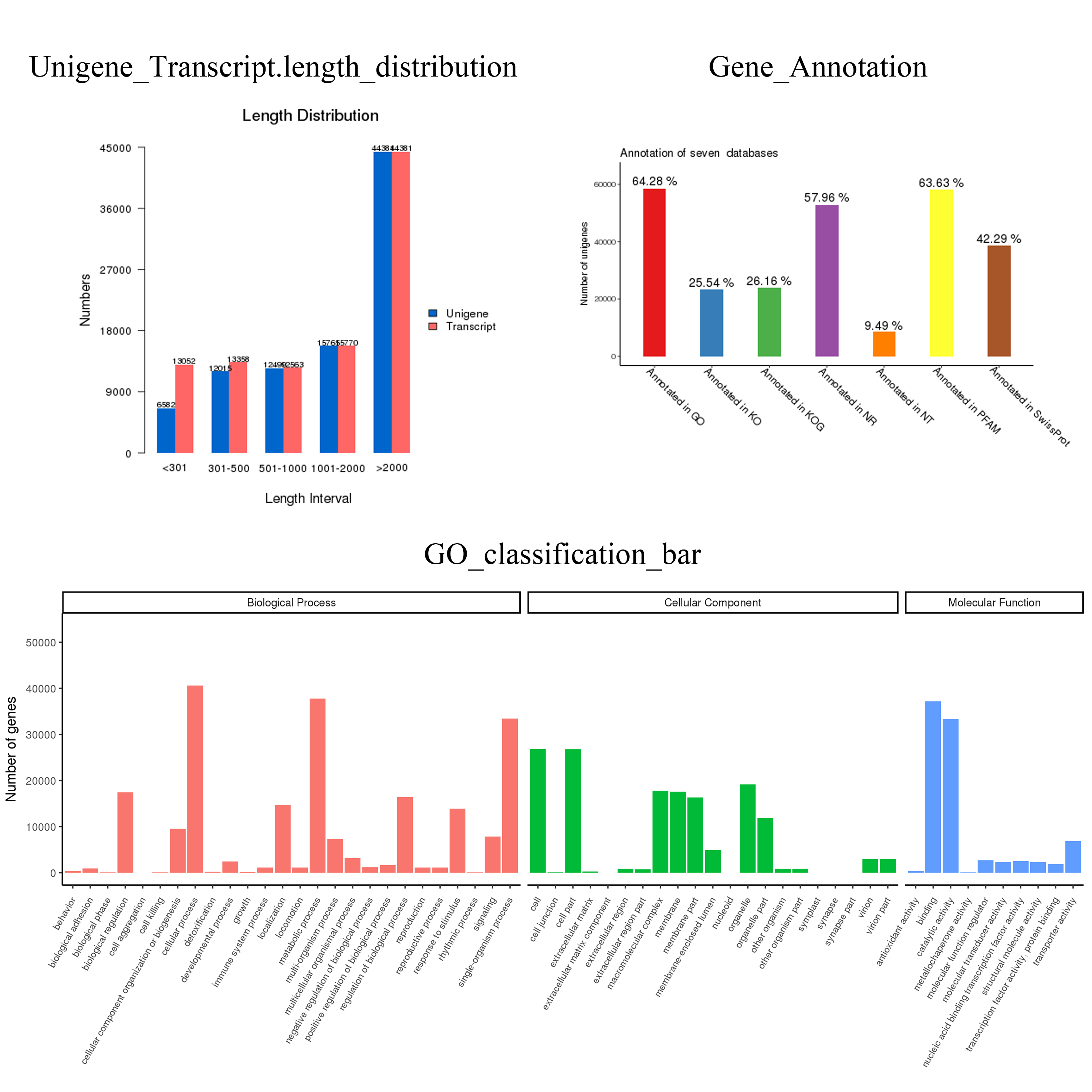

Supplement: Supplementary file 1 [file ijms-19-03359-s001.zip › ijms-365665 - supplementary/Figure S1 The assembled transcriptome information of C. reinhardtii.tif]

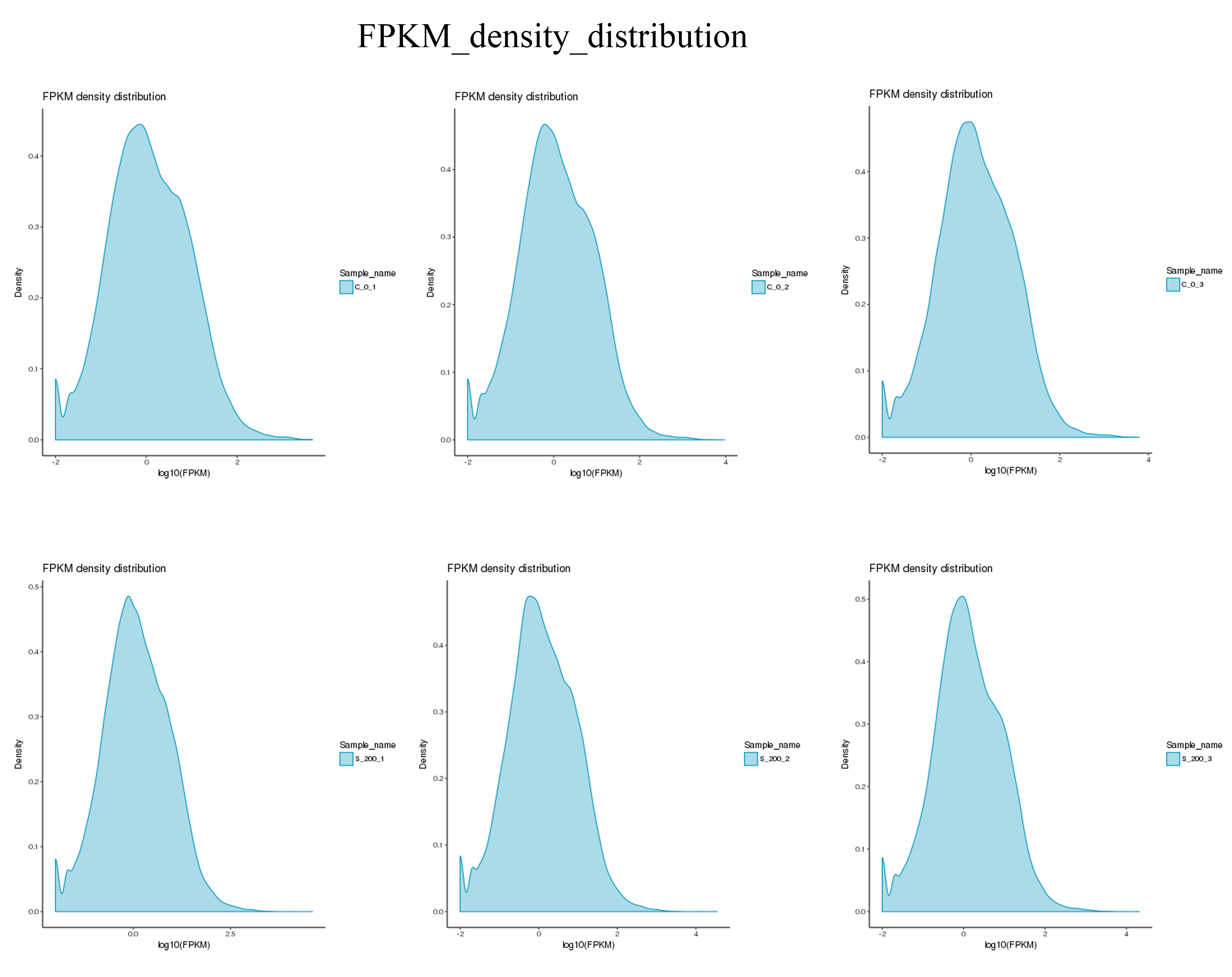

Supplement: Supplementary file 1 [file ijms-19-03359-s001.zip › ijms-365665 - supplementary/Figure S2. The FPKM density distribution of C. reinhardtii.tif]
